# Terahertz electromagnetic fences on a graphene surface plasmon polariton platform

**DOI:** 10.1038/s41598-017-03205-x

**Published:** 2017-06-06

**Authors:** Xidong Wu, Xiang Guo

**Affiliations:** College of Information Science & Electronic Engineering, Zhejiang University, Hangzhou, 310027 Zhejiang China

## Abstract

Controlling the loss of graphene can be used in the field of transformation optics. We propose a new concept of electromagnetic fence on a monolayer graphene surface plasmon polariton platform. Using a Dot-Density-Renderer quasicrystal metasurface, we can simulate the absorption of gradient index optics structures. Numerical simulations show that the incident waves to our designed electromagnetic fence are trapped toward the central lines and quickly absorbed by the high-loss region. Two basic types of electromagnetic fence and its composite structures have been designed and analyzed, which exhibit excellent broadband absorbing performances at 8 THz–12 THz. Because of its advantages in controlling the soft-boundary effects and easy manufacturing characteristics, the proposed electromagnetic fence seems very promising for THz–frequency-transformation plasmonics applications.

## Introduction

Because of the ability to overcome the diffraction limit of light in microchip-sized devices, SPPs are considered as one of the most promising candidates for nanophotonic components^1–3^. For this purpose, graphene has been investigated as a one-atom-thick platform for terahertz devices due to its ability to support and control surface plasmon polariton (SPP) surface waves in the terahertz regime^4^. Unlike those in noble metals, SPP surface waves in graphene can be tightly confined to the graphene layer because the real part of the effective index in graphene is much larger than that in free space^5–12^. In addition, graphene’s most unique and remarkable property is that its conductivity and permittivity can be dynamically tuned by controlling the chemical potential^4, 11^.

A number of schemes have been reported to create desired permittivity patterns on graphene flakes at the sub-wavelength scale^13, 14^. Moreover, the large effective index of graphene can efficiently constrain SPP waves around graphene sheet, which presents a variety of possibilities in the design of optical transformation devices, such as modulators^15–18^, filter^19^, cloaks^20^, and absorbers^21–24^. Additionally, transformation plasmonics has opened new avenues towards the realization of on-chip nanophotonic devices^13^. In particular, because of its easy fabrication, the uneven ground plane biased structure can be used to realize various terahertz devices, such as “electromagnetic black holes”^25^ and “beam-scanning planar lenses”^26^. The uneven ground plane below the dielectric spacer support can be used to easily tune the chemical potential of graphene to achieve desired permittivity patterns. However, this scheme is limited by soft-boundary effects^27^, which imply that the designed uneven ground plane pattern cannot be precisely mapped to the required permittivity pattern.

One way to realize graded index metasurface is to use a various ground-graphene distance approach, as reported in ref. 25. Different ground-graphene distances correspond to different chemical potentials, therefore leading to different indexes. However, this is very difficult to fabricate and suffers severe soft-boundary effects, which can lead to unexpectedly deterioration of the intended function. An alternative approach is reported in ref. 21, in which only two different ground-graphene distances are used. One distance corresponds to a background index, while another corresponds to a “dot” index, which is usually much larger than the background index. The required gradient effective index can then be realized by carefully designing the pattern with various dot sizes. In this way, the manufacturing processes can be easily controlled using planar integration techniques. However, too small dot size should be avoided since it will not only increase fabrication difficulties but lead to more apparent soft-boundary effects as well. In this paper, a new kind of Dot-Density-Renderer (DDR) quasicrystal structure is proposed to simulate gradient effective index by design density of equal size dots. This can efficiently reduce the soft-boundary effects. Moreover, due to the dynamic tuning capability of graphene, the DDR structure provides more design flexibility.

The graphene SPP platform has a finite propagation length, which will limit the device size on this platform. Within the platform, interferences between devices should be carefully controlled by constructing isolating structures. In this paper, a flexible electromagnetic (EM) fence is proposed to trap and absorb the interferences from other devices. Hence, the entire graphene SPP platform can be divided into different operation regions for different devices. Using the designed graded-index structure, both sides of the EM fence can trap and absorb EM waves. Unlike the EM black hole structure reported in ref. 28, the proposed EM fence has no core region. This can be attributed to the large imaginary part of the effective index of the dots region. Definitely, this feature enables an efficient reduction of the EM fence size.

## Result

### Theoretical analysis of the EM fence

**Figure 1:**
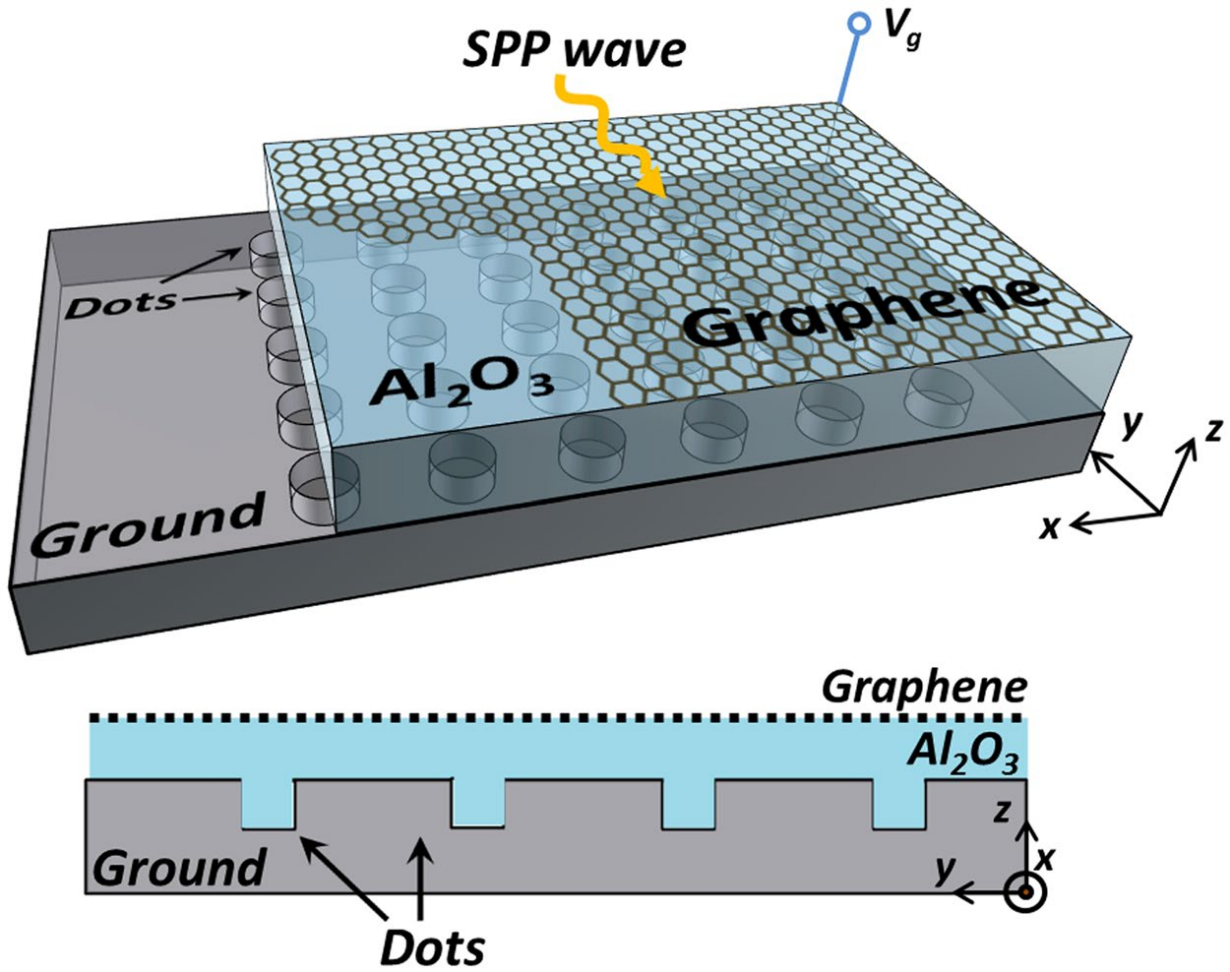
The schematic illustration of the monolayer graphene salient dots biased structure.

In this paper, a Dot-Density-Renderer (DDR) structure is used to build a quasicrystal SPP platform. As shown in Fig. 1, the photonic crystal platform is composed of an uneven bottom ground and a substrate with a monolayer graphene sheet on top. Salient substrate dots with equal diameters and heights are periodically etched and grown inside the ground. When a static voltage bias is applied between the graphene sheet and the uneven ground, it will create two different electric fields on the graphene sheet due to the different voltage gate distances for the dot area and the background area. As a result, two different chemical potentials will be generated, thus yielding two different equivalent complex permittivities for these two different areas. The relationship between the control voltage *V*_*g*_ and the SPP wave number *β* can be easily derived based on the Kubo formula, as given in *Method*. Notably, salient dots with a constant size are used here to relax the requirement for fabrication tolerances^21^ and minimize soft-boundary effects^27^. Clearly, the effective index will mainly depend on the dot density, which will be used to realize the graded-index materials and guided SPP waves on the graphene platform. In this paper, an Al_2_O_3_ substrate with a relative permittivity of 3.2 is selected as the dielectric material.

**Figure 2:**
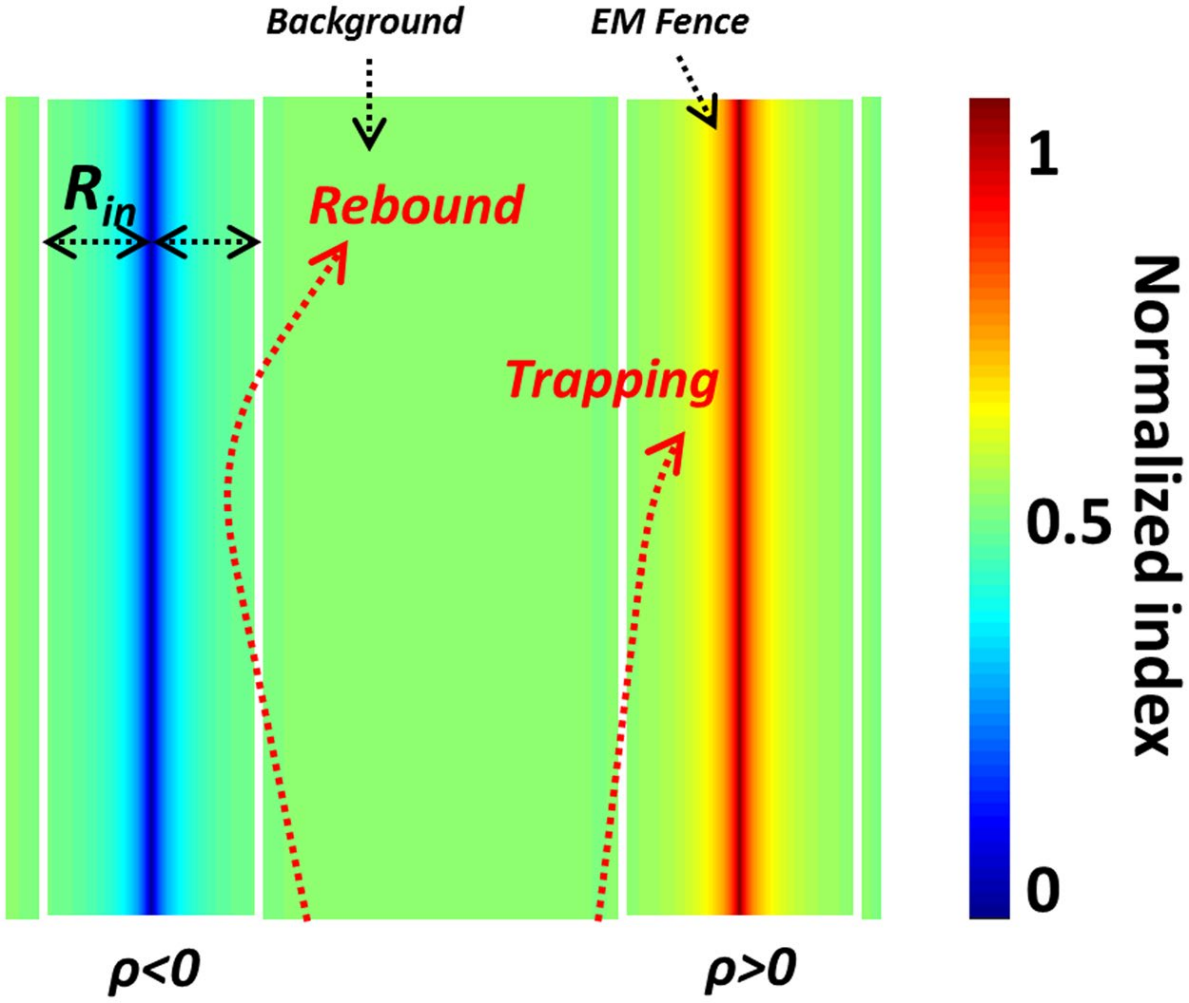
The schematic illustration of the influence of *ρ* on trapping performance.

Figure 2 illustrates a concept of strip-shaped EM fence operating at terahertz bands based on the DDR structure. As shown in Fig. 2, the proposed strip EM fence has two absorbing regions, which can trap and absorb the SPP incident waves that approach from both sides. In order to ensure trapping of EM waves, the graded effective index of the EM fence *n*_*eff*_ should satisfy the expression in Eq. (1)^29–31^.

1 $${n}_{eff}(r)=\{\begin{array}{cc}{n}_{0} & r > {R}_{in}\\ {n}_{0}{(\frac{{R}_{in}}{r})}^{\rho } & r\le {R}_{in}\end{array}$$

where *R*_*in*_ is the half-width of the fence, *r* represents the distance from the central line of the fence, *n*_*0*_ is the background index. It should be noted that the power index *ρ* should be greater than zero to enable trapping of EM waves towards the central line of the EM fence, as shown on the right side of Fig. 2. As discussed in ref. 29, the higher power index *ρ*, the better trapping capability the EM fence can achieve. Alternatively, if we choose a negative value for *ρ*, the SPP incident waves will rebound away from the fence, as shown on the left side of Fig. 2.

The reported EM black holes^29^ usually require shell regions to trap the incident waves into the absorbing core region. However, because the dot regions in our DDR structure can be easily designed to exhibit a lossy characteristic, i.e. a large imaginary part of effective index, which act like the lossy core in ref. 29, SPP incident waves can then be absorbed while travelling through the dot regions of the EM fence. Therefore, the power index *ρ* in Eq. (1) does not need to be greater than 2 as required in refs 29–31. This will clearly bring us more freedom in practical designs.

The most significant feature of the proposed DDR structure is its easiness to simulate the graded index *n*_*eff*_ by carefully designing the dot density. To guarantee penetration of EM waves into the designed EM fence, one should ensure that the operating frequency has not fallen into the forbidden bands of the 2D photonic crystal structure. Since the EM fence does not have a regular lattice distribution, its band structure cannot be easily calculated using analytical methods. Therefore, the distribution of dots should be carefully arranged in a practical design to minimize unwanted reflections at the design frequency band.

**Figure 3:**
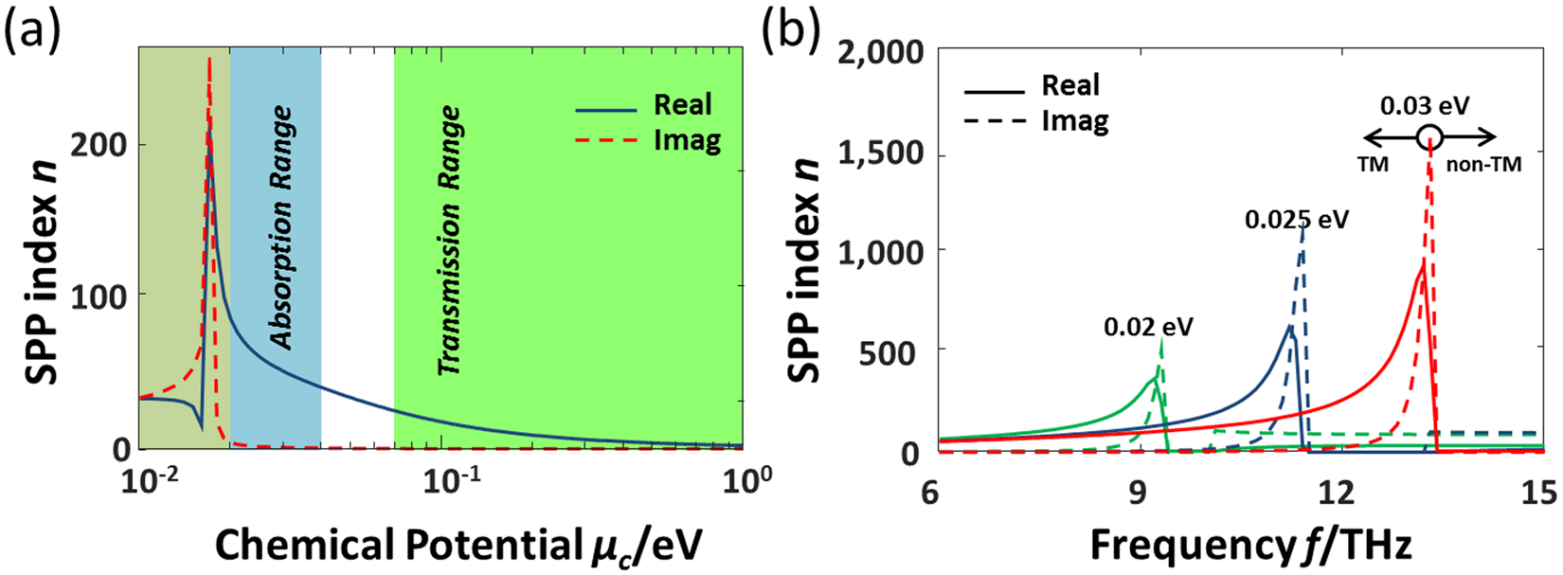
(**a**) SPP index as a function of chemical potential. The curves are calculated at *f* = 8 THz, *τ* = 0.3 ps and *T* = 300 K. (**b**) SPP index as a function of frequency at *μ*_*c*_ = 0.02, 0.025 and 0.03 eV, *τ* = 0.3 ps and *T* = 300 K.

For better absorbing performance, a high imaginary part of the effective index is preferred for the proposed EM fence. For this purpose, the dots region in the DDR structure should be designed to operate at the absorption range, which corresponds to a large *n*_*dot*_ as shown in Fig. 3a. The background region should be designed to fall in the transmission range to ensure transmission of SPP waves, corresponding to a small *n*_*0*_. However, there is a limit to the extent that *n*_*dot*_ can be increased, as the large contrast between *n*_*dot*_ and *n*_*0*_ will result in a sparse dot distribution of the DDR structure. This will clearly increase possibilities of unwanted reflections, thus damaging the trapping capability. Another reason to avoid too large *n*_*dot*_ is that it will experience a steep *n*_*r*_ curve as shown in Fig. 3a, which implies a more sensitive voltage control of the DDR structure. Figure 3b gives the calculated complex index as a function of frequency for three typical chemical potentials. We can then define the cut-off frequency under which the TM mode can be supported. For example, the cut-off frequency for 0.03 eV chemical potential can be estimated as about 13.2 THz. However, strong dispersion will be experienced when the operating frequency is close to the cut-off frequency. Therefore, we will investigate the EM fence under 12 THz in this design.

### Structure and design of the EM fence

#### Black strip

**Figure 4:**
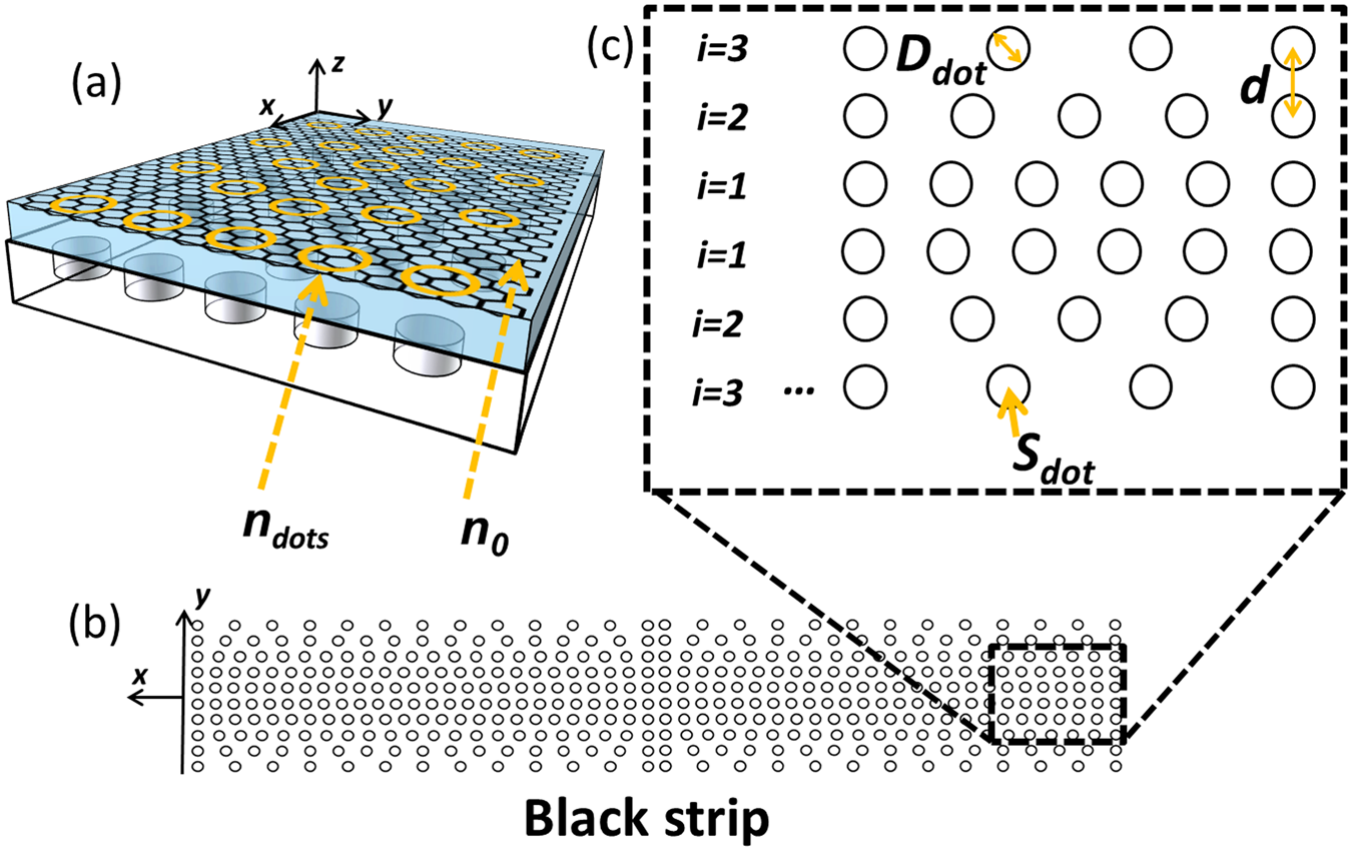
(**a**) Illustration of index *n*_*dot*_ and *n*_*0*_ for dots region and background region, respectively. (**b**) Top-view of the black strip on graphene metasurface. (**c**) The schematic of dot distribution for the black strip.

Figure 4 shows the proposed design of a black strip based on the DDR structure. The effective index of the *i*-th row can be approximated by Eq. (2), where *S*_*dot*_ is the area of a single dot, and *d* represents the distance between two adjacent rows. By substituting Eq. (2) into Eq. (1), the number of dots per unit length of the *i*-th row *N*_*dot*_ can then be easily obtained.

2 $${n}_{eff}(i)=\frac{{n}_{dot}{S}_{dot}{N}_{dot}(i)+{n}_{0}(d-{S}_{dot}{N}_{dot}(i))}{d}$$

**Figure 5:**
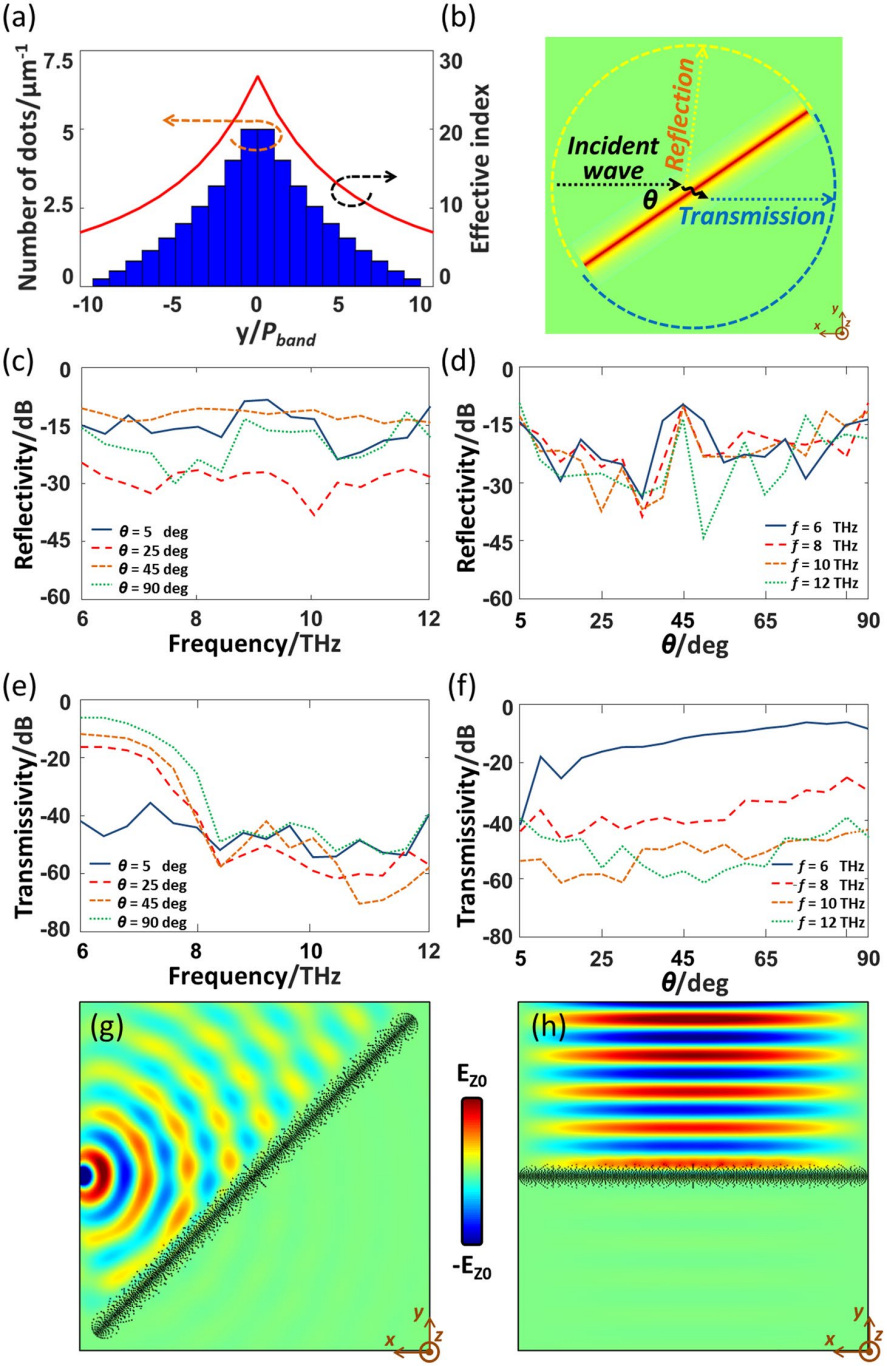
(**a**) The dot density distribution of the designed black strip and the corresponding effective index. (**b**) Illustration of reflection and transmission with incident angle *θ*. (**c**,**d**) The reflectivity as a function of frequency and incident angle. (**e**,**f**) The transmissivity as a function of frequency and incident angle. (**g**,**h**) Field distributions of the black strip illuminated with a point source and a line source at 8 THz.

In this design, the chemical potentials of the dots and background are chosen to be 0.03 eV and 0.3 eV, respectively. The corresponding index can then be calculated as *n*_*dot*_ = 64.24 + 1.40j and *n*_*0*_ = 7.67 + 0.09j at 8 THz. Other design parameters are gives as *ρ* = 1.45, the diameter of dots *D*_*dot*_ = 100 nm, the distance between two adjacent row *d* = 200 nm. The designed black strip is composed of 20 rows, 10 rows each side, with a total width of 60 µm. The calculated number of dots per unit length for each row and the corresponding effective index are shown in Fig. 5a. It should be noted that the resulting effective index will be slightly changed for different frequencies. However, the distribution of the effective index will be quite similar, leading to a similar trapping capability.

To validate the absorbing performance of the designed black strip, the finite element method (FEM) software COMSOL is employed to analyze the scattering. For this purpose, a fitting method is used to describe index *n*_*dot*_ and *n*_*0*_ as function of frequency in COMSOL simulations without need of complicated numerical analysis.

The simulated transmissivity and reflectivity of the designed black strip are shown in Fig. 5c–f as a function of frequency *f* and incident angle *θ*. As shown, the reflectivity is better than −10 dB between 6 THz and 12 THz for incident angles from 5 deg to 90 deg. Apparently, the residual reflections are from the unwanted scattering effects of the edge dots of the designed black strip. The transmissivity is found to be better than −30 dB between 8 THz and 12 THz for most of incident angles. Theoretically, the absorption increases with frequencies, this is because that the imaginary part of the index for the dots region becomes larger for higher frequencies, as shown in Fig. 3b. In conclusion, the designed black strip works satisfactorily within a frequency range between 8 THz and 12 THz.

Figure 5g,h illustrates the field distributions of the designed black strip at 8 THz with two different illumination sources, i.e. a point source in Fig. 5g and a line source in Fig. 5h. It can be clearly observed in both cases that the incident SPP waves are trapped towards the central line of the black strip and get well absorbed. However, noticeable reflections can be observed with the incident angle of 45 deg in Fig. 5g. This quite agrees with our simulations in Fig. 5d, in which the reflection achieves its maximum at incident angle of 45 deg. Moreover, the difficulty in observing the transmitted waves indicates that the transmissivity is very small. This phenomenon indicates a strong absorption behavior of the designed EM fence has been achieved.

#### Black Ring

To demonstrate the flexibility of the EM fence, a black ring structure is introduced, in which the effective index of each ring is designed to imitate the gradient index material. Similarly, the number of dots *N*_*dot*_(*i*) can be calculated according to Eq. (2). For this purpose, an effective index distribution as function of the radius *r* is given in Eq. (3). To simplify the design, *R*_*in*_ is selected as the asymptotic line of the outer ring, and *R*_*out*_ is selected as the asymptote line of the inner ring.

3 $${n}_{eff}(r)=\{\begin{array}{cc}{n}_{0} & 0\le r < {R}_{in}\\ {n}_{0}{(\frac{{R}_{out}-{R}_{in}}{{R}_{out}-r})}^{\rho } & {R}_{in}\le r < \frac{{R}_{out}+{R}_{in}}{2}\\ {n}_{0}{(\frac{{R}_{out}-{R}_{in}}{r-{R}_{in}})}^{\rho } & \frac{{R}_{out}+{R}_{in}}{2}\le r < {R}_{out}\\ {n}_{0} & {R}_{out}\le r\end{array}$$

**Figure 6:**
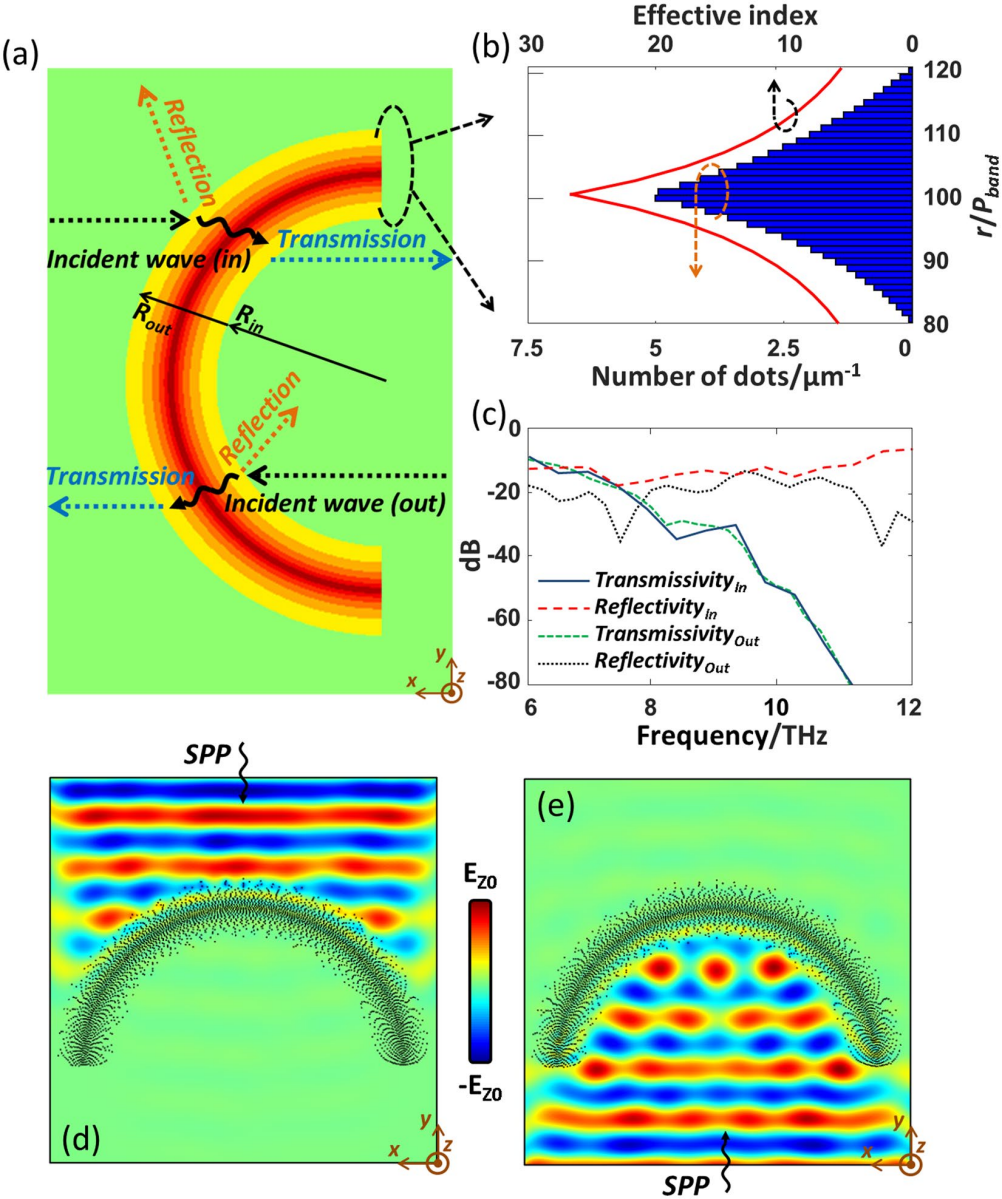
(**a**) Illustration of reflections and transmissions with inward and outward incident waves. (**b**) The dot density distribution of the designed black ring and the corresponding effective index. (**c**) The simulated transmissivities and reflectivities with inward and outward incident waves. (**d**,**e**) Field distributions of the black semi-ring illuminated with inward and outward incident waves at 8 THz.

For the black ring, the chemical potential of the dot and the background are chosen to be 0.03 eV and 0.5 eV, respectively. The corresponding index can then be calculated as *n*_*dot*_ = 64.24 + 1.40j and *n*_*0*_ = 4.66 + 0.05j. Same as the black strip, the diameter of dots is selected as *D*_*dot*_ = 100 nm, and the distance between two adjacent rows *d* = 200 nm. Other design parameters used in Eq. (3) are chosen as *ρ* = 2.04, *R*_*in*_ = 16 µm and *R*_*out*_ = 24 µm. The calculated number of dots per unit length and the corresponding effective index are shown in Fig. 6b.

Figure 6c shows the simulated transmissivity and reflectivity of the designed semi-ring illuminated from two sides of the semi-ring, as marked in Fig. 6a. Similar to the black strip, a transmissivity of less than −30 dB is observed for incidence from both sides, when the frequency is larger than 8 THz. The corresponding reflectivity is better than −10 dB. Figure 6d,e shows the electric field distribution of the designed black-ring at 8 THz, in which good absorption performance is observed. The same conclusion can be made that good fence performance can be achieved for the designed black ring for frequencies between 8 THz to 12 THz.

#### Black complex

**Figure 7:**
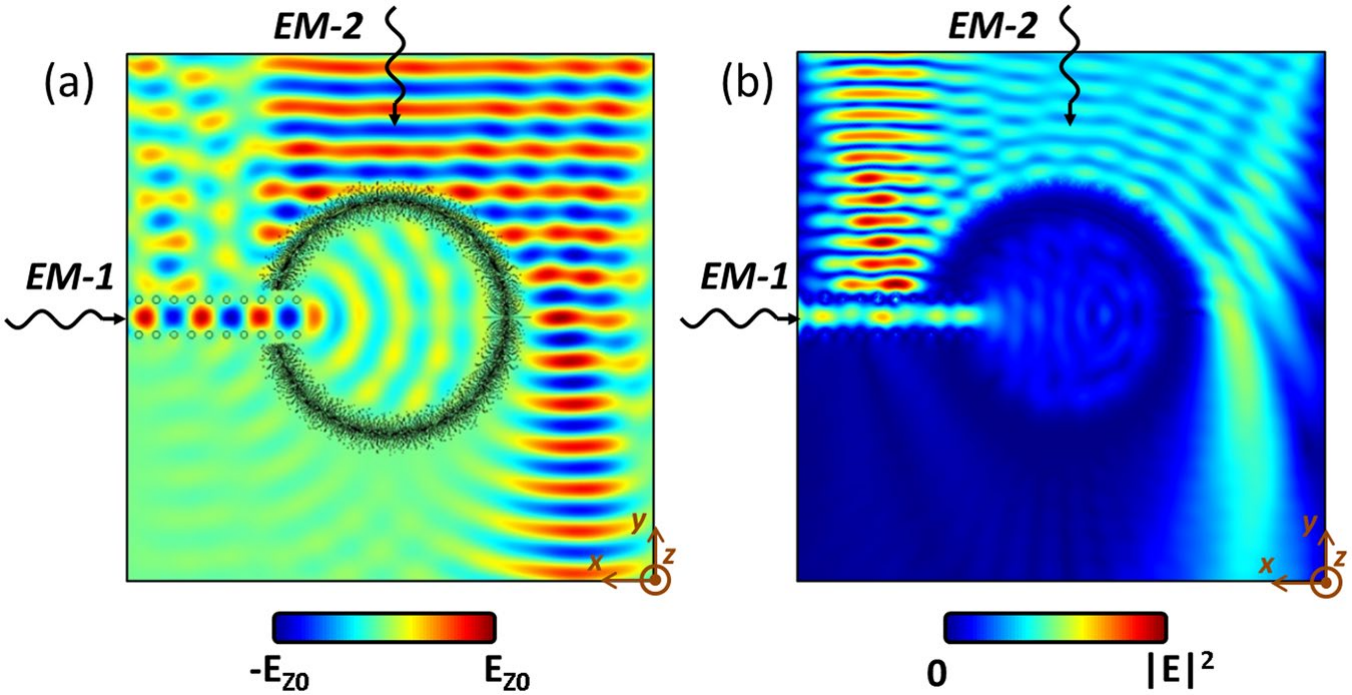
(**a**) Field distribution of black complex with two incident waves *EM-1* and *EM-2* at 8 THz. (**b**) Amplitude distribution.

To demonstrate the performance of the designed EM fence structures, a couple of examples are given in this section. Figure 7 shows a black ring with an open end. The incident SPP wave *EM-1* travels along a single-line-defect photonic crystal waveguide and then couples into the black ring. At the same time, a line source of SPP wave *EM-2* illuminates the black ring from the outside. Figure 7a shows a snapshot of the electric fields simultaneously illuminated by SPP waves *EM-1* and *EM-2*. For *EM-1*, a good travelling wave characteristic can be observed inside the photonic crystal waveguide. Also, a cylindrical phase front is quite obvious inside the black ring, which indicates there are very few reflections caused by the black ring. For *EM-2*, however, much stronger reflections can be seen from the single-line-defect photonic crystal waveguide than from the black ring. Furthermore, it can be seen that the *EM-1* and *EM-2* are well isolated by the designed black ring, which validates the good performance of the EM fence. This is more obvious when comparing the waves inside and outside of the black ring in Fig. 7b, which shows the amplitude distribution of the electric fields from COMSOL simulations. Also, higher contrast fringes can be observed from the photonic crystal waveguide, which indicates much stronger reflections are experienced.

**Figure 8:**
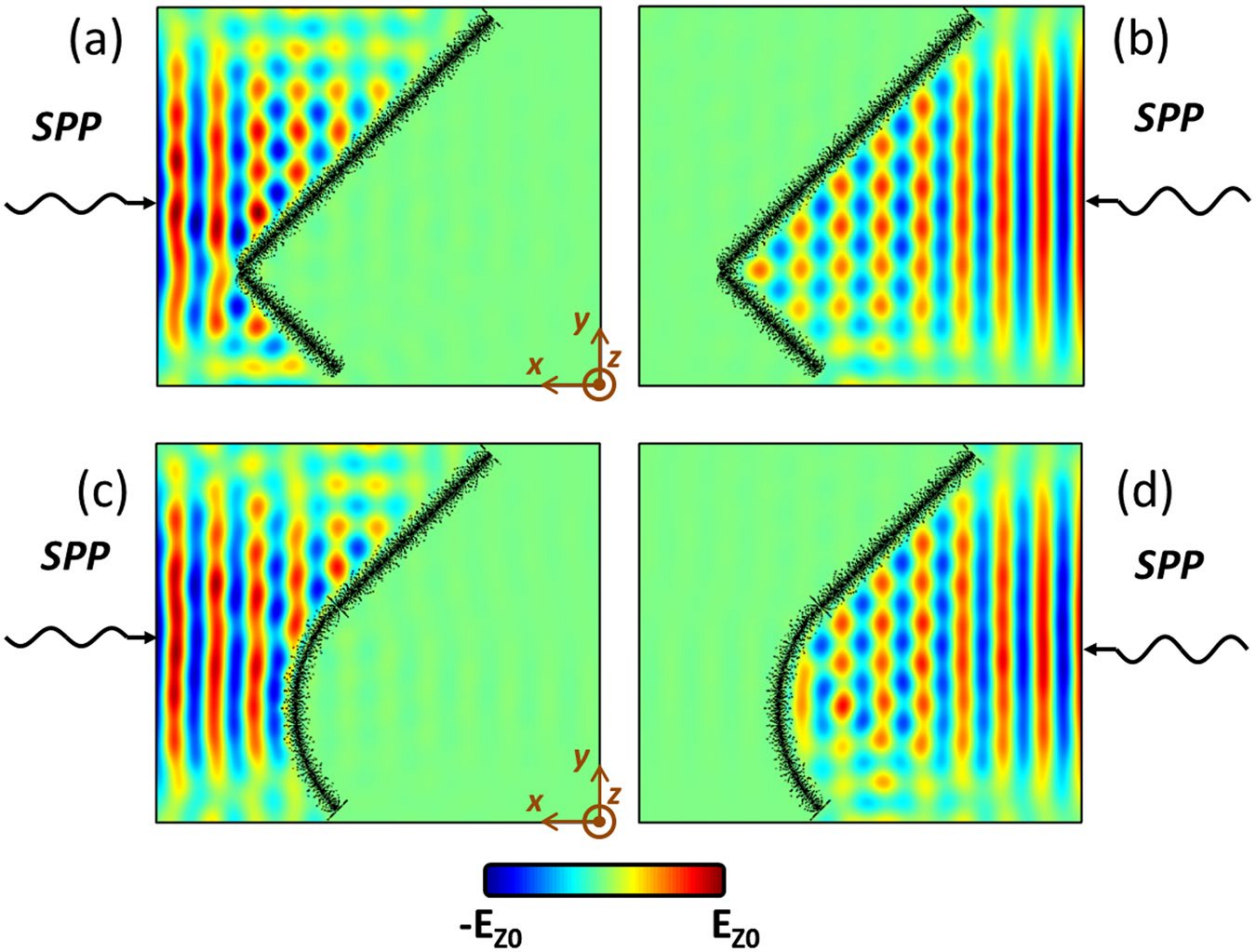
Field distributions of composite structures of the EM fence at 8 THz. (**a**,**b**) A right-angle corner composite EM fence. (**c**,**d**) A rounded-corner composite EM fence.

For more flexible designs, the strip and ring structures can be combined together for better performances. Figure 8 shows another example of composite structure, in which a black strip is right angle bended in the middle. As expected, appreciable reflections can be observed from the right-angle corner for both left-side incidence and right-side incidence, as shown in Fig. 8a,b. The reflections can be somewhat reduced by using a quarter length of black ring to build EM fence with a rounded corner, as shown in Fig. 8c,d.

## Conclusion

In summary, we theoretically realize a concept of electromagnetic fence based on Dot-Density-Renderer quasi-photonic crystal structure, which is built on a monolayer graphene SPP platform with an uneven ground bias. Since all dots are in the same diameter, the soft-boundary effects can be effectively contained when an uneven ground structure is used to bias the graphene sheet. Two types of electromagnetic fences are designed, i.e. black strip and black ring, which exhibit quite favorable absorption properties over a very wide frequency band for incident waves over a large range of angles. Furthermore, the composite structures enable the realization of more flexible fence applications in the field of transformational plasmon optics.

## Method

### SPP waves on a graphene platform

The surface conductivity of graphene can be modeled using the Kubo formula:

4 $$\sigma ={\sigma }_{intra}+{\sigma }_{inter}$$

where *σ*_*intra*_ is due to the intraband contribution, and *σ*_*inter*_ is due to the interband contribution.

5 $${\sigma }_{intra}(\omega )=-j\frac{{e}^{2}{k}_{B}T}{\pi {\hslash }^{2}(\omega -j{\tau }^{-1})}[\frac{{\mu }_{c}}{{k}_{B}T}+2\,\mathrm{ln}({e}^{-{\mu }_{c}/{k}_{B}T}+1)]$$

6 $${\sigma }_{inter}(\omega )=\frac{-j{e}^{2}}{4\pi \hslash }\,\mathrm{ln}(\frac{2|{\mu }_{c}|-(\omega -j{\tau }^{-1})\hslash }{2|{\mu }_{c}|+(\omega -j{\tau }^{-1})\hslash })$$

where *k*_*B*_ is the Boltzmann’s constant, *e* is the charge of an electron, and *ћ* is the reduced Planck’s constant. The Kubo formula shows that the surface conductivity of graphene depends on the frequency *ω*, scattering rate *τ*, temperature *T*, and chemical potential *μ*_*c*_. The scattering rate *τ* is given by ref. 32:

7 $$\tau =\frac{\mu \hslash \sqrt{\pi n}}{e|{\nu }_{F}|}$$

where |*v*_*F*_| = 1.1 × 10^6^ m/s is the Fermi velocity, *n* is the carrier density, and *μ* is the mobility. Because the mobility *μ* of CVD graphene grown on hexagonal-boron nitride can easily approach a value of up to 60000 cm^2^/(Vs)^33^, a choice of *τ* = 0.3 ps is an appropriate assumption for our device simulation, according to Eq. (7).

In general, the surface conductivity of graphene is a complex number. According to Maxwell’s Equations, the complex conductivity of graphene can be used to obtain the equivalent complex permittivity of a very thin graphene sheet. Assuming a thickness *Δ* of the graphene sheet, we can obtain the following relations:

8 $$\varepsilon =\frac{-{\sigma }_{i}}{\omega {\rm{\Delta }}}+{\varepsilon }_{0}+j\frac{{\sigma }_{r}}{\omega {\rm{\Delta }}}.$$

9 $${\varepsilon }_{r}=\frac{-\,{\sigma }_{i}}{\omega {\rm{\Delta }}}+{\varepsilon }_{0}\approx \frac{-{\sigma }_{i}}{\omega {\rm{\Delta }}}.\quad \quad {\varepsilon }_{i}=j\frac{{\sigma }_{r}}{\omega {\rm{\Delta }}}.$$

Therefore, when the complex conductivity of a *Δ*-thick graphene sheet exhibits a positive imaginary part, it effectively behaves as a very thin “metal” sheet. A transverse-magnetic (TM) SPP surface wave can subsequently be supported on this sheet, and the guided mode wave number *β* can be expressed by Eq. (10) as *Δ* → 0.

10 $$\beta ={k}_{0}\sqrt{1-\frac{2}{{\eta }_{0}\sigma }}.$$

Here, *η*_*0*_ is the free-space wave impedance, and *k*_*0*_ is the free-space wave number. Notably, the wave number *β* is a complex number because the conductivity *σ* is complex. For SPP waves, the effective propagation length can be defined as 1/Im(*β*). The graded-index structure can be designed by modifying the real part of *β*.

For an isolated graphene sheet, the chemical potential *μ*_*c*_ can be determined by the carrier density *n* using the following equation:

11 $$n=\frac{2}{\pi {\hslash }^{2}{v}_{F}^{2}}[{({k}_{B}T)}^{2}{\int }_{-{\mu }_{c}/{k}_{B}T}^{{\mu }_{c}/{k}_{B}T}\frac{x}{{e}^{x}+1}dx+{k}_{B}T{\mu }_{c}\,\mathrm{ln}({e}^{-{\mu }_{c}/{k}_{B}T}+1)+{k}_{B}T{\mu }_{c}\,\mathrm{ln}({e}^{{\mu }_{c}/{k}_{B}T}+1)]$$

Applying a voltage *V*_*g*_ on the gate can modulate the carrier density of graphene through the following equation:

12 $${V}_{g}=\frac{\hslash |{\nu }_{F}|\sqrt{\pi n}}{e}+\frac{ne}{{C}_{g}}.$$

where *C*_*g*_ = *ε*/*t* is the geometric capacitance, *ε* is the permittivity of the dielectric material, and *t* is the dielectric layer thickness, which may be the key factor for controlling the electrical characteristics of graphene in practical design. It should be noted that the mode area of monolayer graphene sheet waveguides is extremely small^34^ (approximately 10^−7^λ_0_^2^). In other words, the distance between the graphene sheet and the uneven ground is large enough to ensure that the guided mode of the SPP waves will not be affected.

## References

[1] Barnes WL, Dereux A, Ebbesen TW (2003). Surface Plasmon Subwavelength Optics. Nature..

[2] Gramotnev DK, Bozhevolnyi SI (2010). Plasmonics Beyond the Diffraction Limit. Nat. Photon..

[3] Zhu Y, Hu X, Yang H, Gong Q (2014). On-Chip Plasmon-Induced Transparency Based On Plasmonic Coupled Nanocavities. Sci. Rep..

[4] Vakil A, Engheta N (2011). Transformation Optics Using Graphene. Science..

[5] Ju L, Geng B, Horng J, Girit C, Martin M (2011). Graphene Plasmonics for Tunable Terahertz Metamaterials. Nat. Nanotechnol..

[6] Gumbs G, Iurov A, Wu J, Lin MF, Fekete P (2016). Plasmon Excitations of Multi-layer Graphene On a Conducting Substrate. Sci. Rep..

[7] Dai X, Jiang L, Xiang Y (2015). Low Threshold Optical Bistability at Terahertz Frequencies with Graphene Surface Plasmons. Sci. Rep..

[8] Dubrovkin AM, Tao J, Chao YX, Zheludev NI, Jie WQ (2015). The Reduction of Surface Plasmon Losses in Quasi-Suspended Graphene. Sci. Rep..

[9] Zhao T, Gong S, Hu M, Zhong R, Liu D (2015). Coherent and Tunable Terahertz Radiation from Graphene Surface Plasmon Polarirons Excited by Cyclotron Electron Beam. Sci. Rep..

[10] Cheng BH, Chen HW, Jen YJ, Lan YC, Tsai DP (2016). Tunable Tapered Waveguide for Efficient Compression of Light to Graphene Surface Plasmons. Sci. Rep..

[11] Hao R, Peng XL, Li EP, Xu Y, Jin JM (2015). Improved Slow Light Capacity in Graphene-Based Waveguide. Sci. Rep..

[12] Lu H, Zeng C, Zhang Q, Liu X, Hossain MM (2015). Graphene-Based Active Slow Surface Plasmon Polaritons. Sci. Rep..

[13] Della Valle G, Longhi S (2010). Graded Index Surface-Plasmon-Polariton Devices for Subwavelength Light Management. Phys. Rev. B.

[14] He S, He Y, Jin Y (2012). Revealing the Truth About ‘Trapped Rainbow’ Storage of Light in Metamaterials. Sci. Rep..

[15] Gan X, Shiue RJ, Gao Y, Mak KF, Yao X (2012). High-Contrast Electro-Optic Modulation of a Photonic Crystal Nanocavity by Electrical Gating of Graphene. Nano Lett..

[16] Li W (2014). Ultrafast All-Optical Graphene Modulator. Nano Lett..

[17] Yang L (2015). An All-Optical Modulation Method in Sub-Micron Scale. Sci. Rep..

[18] Matthaiakakis N, Mizuta H, Charlton M (2016). Strong Modulation of Plasmons in Graphene with the Use of an Inverted Pyramid Array Diffraction Grating. Sci. Rep..

[19] Shi B, Cai W, Zhang X, Xiang Y, Zhan Y (2016). Tunable Band-Stop Filters for Graphene Plasmons Based on Periodically Modulated Graphene. Sci. Rep..

[20] Chen P (2011). & Alù, A. Atomically Thin Surface Cloak Using Graphene Monolayers. ACS Nano..

[21] Zeng C, Liu X, Wang G (2014). Electrically Tunable Graphene Plasmonic Quasicrystal Metasurfaces for Transformation Optics. Sci. Rep..

[22] Goykhman I (2016). On-Chip Integrated, Silicon–Graphene Plasmonic Schottky Photodetector with High Responsivity and Avalanche Photogain. Nano Lett..

[23] Liu Y, Zentgraf T, Bartal G, Zhang X (2010). Transformational Plasmon Optics. Nano Lett..

[24] Wang Z, Wang B, Wang K, Long H, Lu P (2016). Vector Plasmonic Lattice Solitons in Nonlinear Graphene-Pair Arrays. Opt. Lett..

[25] Jiang Y, Lu WB, Xu HJ, Dong ZG, Cui TJ (2012). A Planar Electromagnetic “Black Hole” Based On Graphene. Phys. Lett. A.

[26] Ju XH, Bing LW, Jiang Y, Gao Dong Z (2012). Beam-Scanning Planar Lens Based On Graphene. Appl. Phys. Lett..

[27] Forati E, Hanson GW (2014). Soft-Boundary Graphene Nanoribbon Formed by a Graphene Sheet Above a Perturbed Ground Plane. J. Opt..

[28] Cheng Q, Cui TJ, Jiang WX, Cai BG (2010). An Omnidirectional Electromagnetic Absorber Made of Metamaterials. New J. Phys..

[29] Narimanov EE, Kildishev AV (2009). Optical Black Hole: Broadband Omnidirectional Light Absorber. Appl. Phys. Lett..

[30] Kildishev AV, Prokopeva LJ, Narimanov EE (2010). Cylinder Light Concentrator and Absorber: Theoretical Description. Opt. Express..

[31] Liu S (2010). Graded Index Photonic Hole: Analytical and Rigorous Full Wave Solution. Phys. Rev. B.

[32] Christensen J, Manjavacas A, Thongrattanasiri S, Koppens FHL, Javier Garcia De Abajo F (2012). Graphene Plasmon Waveguiding and Hybridization in Individual and Paired Nanoribbons. ACS Nano..

[33] Dean CR (2010). Boron Nitride Substrates for High-Quality Graphene Electronics. Nat. Nanotechnol..

[34] Koppens FHL, Chang DE, García de Abajo FJ (2011). Graphene Plasmonics: A Platform for Strong Light–Matter Interactions. Nano Lett..

